# A Novel Hydrogel-Based 3D In Vitro Tumor Panel of 30 PDX Models Incorporates Tumor, Stromal and Immune Cell Compartments of the TME for the Screening of Oncology and Immuno-Therapies

**DOI:** 10.3390/cells12081145

**Published:** 2023-04-13

**Authors:** Bin Xue, Julia Schüler, Christopher M. Harrod, Kanstantsin Lashuk, Zoji Bomya, Kolin C. Hribar

**Affiliations:** 1 Cypre, Inc., South San Francisco, CA 94080, USA; 2 Charles River Discovery Research Services Germany GmbH, 79108 Freiburg, Germany

**Keywords:** three dimensional models, 3D cell culture, patient-derived xenograft, tumor panel, drug screening, immuno-oncology, immunotherapy, tumor microenvironment

## Abstract

Human-relevant systems that mimic the 3D tumor microenvironment (TME), particularly the complex mechanisms of immuno-modulation in the tumor stroma, in a reproducible and scalable format are of high interest for the drug discovery industry. Here, we describe a novel 3D in vitro tumor panel comprising 30 distinct PDX models covering a range of histotypes and molecular subtypes and cocultured with fibroblasts and PBMCs in planar (flat) extracellular matrix hydrogels to reflect the three compartments of the TME—tumor, stroma, and immune cells. The panel was constructed in a 96-well plate format and assayed tumor size, tumor killing, and T-cell infiltration using high-content image analysis after 4 days of treatment. We screened the panel first against the chemotherapy drug Cisplatin to demonstrate feasibility and robustness, and subsequently assayed immuno-oncology agents Solitomab (CD3/EpCAM bispecific T-cell engager) and the immune checkpoint inhibitors (ICIs) Atezolizumab (anti-PDL1), Nivolumab (anti-PD1) and Ipilimumab (anti-CTLA4). Solitomab displayed a strong response across many PDX models in terms of tumor reduction and killing, allowing for its subsequent use as a positive control for ICIs. Interestingly, Atezolizumab and Nivolumab demonstrated a mild response compared to Ipilimumab in a subset of models from the panel. We later determined that PBMC spatial proximity in the assay setup was important for the PD1 inhibitor, hypothesizing that both duration and concentration of antigen exposure may be critical. The described 30-model panel represents a significant advancement toward screening in vitro models of the tumor microenvironment that include tumor, fibroblast, and immune cell populations in an extracellular matrix hydrogel, with robust and standardized high content image analysis in a planar hydrogel. The platform is aimed at rapidly screening various combinations and novel agents and forming a critical conduit to the clinic, thus accelerating drug discovery for the next generation of therapeutics.

## 1. Introduction

The tumor microenvironment (TME) is a complex and dynamic network of tumor, stromal, and immune cells in an extracellular matrix (ECM) [1,2,3]. The relative composition and spatial orientation (e.g., immune cold, excluded, hot) of these cell types in three dimensions (3D) directs their response or lack thereof to many targeted therapies and immunotherapy [4]. Of note, the immunosuppressive stroma, including cancer-associated fibroblasts, in solid tumors plays a unique role in both supporting cancer growth as well as hindering the migration of cytolytic CD8+ T cells [4,5,6]. The stroma also acts as a physical barrier to large molecule penetration and T-cell migration by virtue of its stiffened ECM [7,8]. In light of these biological complexities, it is paramount that we develop 3D in vitro models that more thoroughly represent the TME biology and may be used to screen the next generation of biotherapeutics. Moreover, the recent passing of the FDA Modernization Act 2.0 in the US Congress (December 2022) as well as the core tenets of the 3Rs implore us to reduce, refine and replace animal models in drug discovery—both for animal welfare and to increase our success rate in subsequent human clinical trials [9].

Historically, in vitro assays have suffered from a lack of biological complexity (such as two-dimensional monolayers on plastic), challenges with assay robustness (e.g., organ-on-chips working at micro or nanoscale volumes [10,11]), and high-throughput analysis. Matrix-free 3D spheroid assays, while enabling more complex attributes of the TME such as hypoxia and proliferation gradients, fail to create a proper stromal barrier surrounding the tumor (instead, fibroblasts will often reside in the interior of the tumor spheroid [12]). T-cell infiltration into spheroids can also be challenging to analyze at high throughput due to optical clarity issues [13]. First-generation matrix-based systems such as collagen and basement membrane extract suffer from batch variability and sometimes growth factor contamination [14] as well as rapid gel degradation or contraction in the presence of fibroblasts [15], complicating their utility in high-throughput drug screening of the TME. Moreover, their incorporation into microfluidic channels enhances the technical challenges with organ-on-a-chip technologies [10]. Thus, a platform that combines more biological complexity of the TME with robustness in setup and scalability in analysis would be greatly beneficial to cancer drug discovery and immunotherapy development.

Based on these needs, we constructed a 3D tumor panel comprising 30 patient-derived xenograft (PDX) in vitro models of various solid tumor indications for screening small and large molecules in a dose-dependent manner. Each model included PDX-derived tumor cell lines, fibroblasts to mimic the stromal compartment, and peripheral blood mononuclear cells (PBMCs) to represent the immune compartment of the TME, all housed in an planar (flat) extracellular matrix hydrogel in 96-well plate format. The gel planarity enabled consistent tumor growth and distribution of PBMCs on the surface of the gel across the duration of the 7–9 day assay. We established standardized and reproducible endpoint analytics from high-content imaging, such as tumor size, tumor killing, and T-cell infiltration, and performed routine cytokine analysis of the supernatants. Ultimately, the platform has the potential to bridge compound discovery and in vivo pharmacology with complex 3D in vitro biology and may serve one day to replace animal models for antitumor determination of various oncology and immunotherapy test articles.

## 2. Materials and Methods

### 2.1. Therapeutic Compounds

Cisplatin, Nivolumab, Atezolizumab, and Ipilimumab were purchased from Selleckchem (Houston, TX, USA). Solitomab was purchased from ProSci Inc. (Poway, CA, USA).

### 2.2. Cultivation of PDX Lines and Fibroblasts In Vitro

PDX-derived cell lines were supplied by Charles River from Charles River’s Cancer Model Database (https://compendium.criver.com/ (accessed on 18 November 2021)) and were grown at 37 °C in a humidified atmosphere with 5% CO_2_ in RPMI 1640 medium supplemented with 10% (*v*/*v*) fetal bovine serum and 100 U/mL Penicillin-Streptomycin. PDX cells were used within 10 passages and are listed in Table 1 [16].

Human dermal fibroblasts (HDF, ScienCell Research Laboratories, Carlsbad, CA, USA) were grown at 37 °C in a humidified atmosphere with 5% CO_2_ in Fibroblast growth medium 3 supplemented with 10% fetal bovine serum, 100 U/mL Penicillin-Streptomycin, 1 ng/mL basic fibroblast growth factor and 5 μg/mL insulin (PromoCell, Heidelberg, Germany).

### 2.3. PDX In Vitro Model Setup in the VersaGel Hydrogel Platform

Cells were trypsinized and counted. A mixture of PDX at 0.5 million per milliliter and HDF cells at 0.4 million per milliliter (corresponding to 1:0.8 cell ratio) was combined with the biocompatible hydrogel VersaGel [17] in a 1:1 ratio (media to gel), pipetted into 96-well plates at 40 μL per well, and crosslinked into flat, thin layers (less than 1 mm thickness) in 96-well microplates for 70 s [17]. In some cases, the models required higher starting tumor density of 1.0 million per milliliter for proper cell growth.

VersaGel is a photo-crosslinkable, microporous bio-polymer that allows for integrin binding and MMP degradation; moreover, the hydrogel can be molded into flat, thin shapes in microwell plates using a custom light exposure and gel molding platform. The fully constructed 3D cell model was then grown in RPMI-1640 medium supplemented with 10% (*v*/*v*) fetal bovine serum (FBS), 100 U/mL Penicillin-Streptomycin, 1 ng/mL basic fibroblast growth factor (bFGF) and 5 μg/mL insulin. The cells were cultured for 3 to 5 days to form tumorspheres before the addition of PBMCs and testing compounds.

### 2.4. Drug Dosing and PBMC Addition

Cisplatin was dissolved in diH_2_O, while Solitomab, Atezolizumab, Nivolumab, and Ipilimumab were solubilized by the manufacturer. Cisplatin (200 µM high dose, 1:4 dilution, diH_2_O vehicle) and Solitomab (625 µg/mL high dose, 1:4 dilution, PBS vehicle) compounds were administered to 3D tumor models on day 3–5 after 3D setup in a 6-dose, serially diluted fashion in duplicate. For Cisplatin, the oncology panel (PDX + HDF, without PBMCs) was utilized, while for Solitomab, PBMCs were added on the same day as compound treatment. PBMCs were supplied by Charles River (Freiburg, Germany) for the Solitomab treatment. For ICIs, PBMCs were purchased from Hemacare (Northridge, CA, USA). PBMCs were thawed, counted, and added to the 3D models at 50,000 cells per well in RPMI-1640 media supplemented with 10% (*v*/*v*) FBS, 100 U/mL Penicillin-Streptomycin, and 10 U/mL recombinant human Interleukin-2 (IL-2, STEMCELL Technologies, Vancouver, BC, Canada). Atezolizumab, Nivolumab, and Ipilimumab were administered to a subset of models on the Panel in a 3-dose, serially diluted fashion (1, 10, 100 µg/mL, PBS vehicle) and compared to Solitomab as the positive control (100 µg/mL). In a follow-up study, PBMCs from the same donor (from Hemacare) were either co-embedded with PDX and fibroblasts inside the hydrogel during 3D setup or directly after 3D setup on the hydrogel surface to compare methods of co-culture in their response to Nivolumab in 6-dose format (100 µg/mL high dose, 1:4 serial dilution, PBS vehicle) over the standard treatment time of 4 days.

### 2.5. High-Content Image Analysis

Tumorspheres were stained with Hoechst nuclear dye and DRAQ7 dead cell dye, followed by fixation with 4% paraformaldehyde. They were subsequently imaged using ImageXpress Micro Confocal High-Content imaging system (IXMC, Molecular Devices, San Jose, CA, USA).

Tumorsphere size, total tumor area (tumor count multiplied by the average tumor size) and tumor cell death were quantitated with the MetaXpress software (Molecular Devices) and proprietary image analysis algorithms. Tumorspheres were identified using a masking approach with the Hoechst nuclear stain. DRAQ7 staining assessed the dead tumor cells as a percentage within tumorspheres.

### 2.6. Calculation of Drug Response Curves

IC50 curves were calculated using the R nplr package [18]. The curves were plotted using log10 form of drug concentration as x-axis and the proportion of tumor growth or tumor killing normalized to the untreated condition as y-axis. Vehicle controls were included in the curve by replacing concentration with 10 × 10^−11^. The logistic regression parameters were automatically selected to fit the curve. E_max_/E_0_ were calculated from the IC50 maximum and minimum values for each of the three values—tumor size, total tumor area, and tumor death. EpCAM expression was sourced per PDX model in Charles River’s Cancer Model Database and compared to E_max_/E_0_ responses for Solitomab.

### 2.7. Multiplex Cytokine Analysis

The supernatant was collected and snap-frozen after completion of the 3D in vitro culture. The supernatants were then thawed and subjected to cytokine analysis using the Cytokine/Chemokine/Growth Factor Convenience 45-Plex Human ProcartaPlex™ Panel 1 (#EPXR450-12171-901, ThermoFisher, Waltham, MA, USA) following the manufacturer’s instructions. Wells were pooled and measured in duplicate. The samples were incubated with magnetic beads for 2 h. Plates were washed manually with the Bio-Plex^®^ handheld magnetic washer (#171020100, BioRad, Hercules, CA, USA), then measured using a Bio-Plex^®^ 200 System (#171000201, BioRad, Hercules, CA, USA).

### 2.8. Immunofluorescence Staining

Fixed tumorspheres were permeabilized with 0.1% Triton X-100 for 30 min. The cells were then stained with 5 μg/mL of primary antibody overnight at 4 °C and 1 μg/mL of Alexa Fluor 568-conjugated goat anti-mouse IgG (H + L) secondary antibody (ThermoFisher) at room temperature for 1 h. Z-stack confocal images (10×) were captured and analyzed using high-content imaging. Mouse anti-human CD45 primary antibody (CD45-2B11, eBioscience™, San Diego, CA, USA), FITC-conjugated CD3 antibody (UCHT1, eBioscience™), mouse anti-human CD8a antibody (RPA-T8, eBioscience™, San Diego, CA, USA) and PE CF594-conjugated CD163 (GHI/61, BD) were used in the experiments.

## 3. Results

### 3.1. Setup in Cypre 3D Platform and Selection of 30 PDX Panel

Here, we developed a comprehensive 3D tumor panel in a 96-well plate format that covers a range of tumor indications and molecular subtypes to expand the application of VersaGel to high-throughput cancer drug screening. We selected 30 PDX lines from Charles River’s Cancer Model Database across solid tumor indications including bladder, colorectal, gastric, head neck, liver, non-small cell lung cancer (NSCLC), breast, melanoma, ovarian, pancreatic, mesothelioma, renal, sarcoma and uterus (Table 1).

The PDX tumors were previously developed by Charles River into low passage in vitro cell lines for scalable cell culture and ultimately repeatable drug screening. Low passage number (<10) of these PDX-derived cell lines was used to preserve some of the heterogeneity of the parental lines. We optimized tumor growth by varying VersaGel density (stiffness and porosity) and cell (tumor, fibroblast) density conditions. It was determined that most models grew in the standard 1:0.8 starting ratio of tumor to fibroblasts, while a subset of models required higher concentrations of one cell type or the other in order to expand effectively.

**Figure 1:**
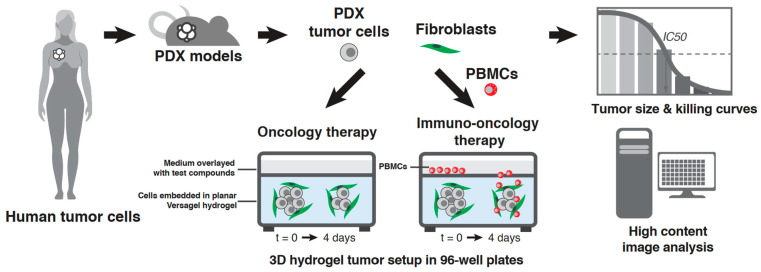
Three-dimensional VersaGel Tumor Model Process: Schematic of the 3D tumor model setup process. First, patient cells are grown in PDX, followed by their establishment into cell lines with limited passages. The PDX-derived cell lines are mixed with human dermal fibroblasts (HDF) and embedded in the VersaGel hydrogel in 96-well plates. Several days later, drug compound and PBMCs are added to the assays, followed by endpoint analysis on day 4 using high-content imaging to analyzing tumor size and killing.

Once the tumor and fibroblast cells were embedded in the hydrogel, we allowed the cells to self-aggregate over 3–5 days and form multicellular tumorspheres with elongated fibroblasts wrapping around and interconnecting the tumorspheres. Following that, we dosed the 3D tumor plates with test compounds for 4 days and optionally with PBMCs for immunotherapy screening. Standard 6-dose, serially diluted, curves were employed for screening of Cisplatin and Solitomab with a positive control (1 µM Staurosporine) and negative control (vehicle), in duplicate (Figure 1).

The 96-well plate format allowed for high-throughput phenotypic screening in a dose-dependent manner. The 3D models were stained at endpoint (7–9 days total of culture time) with Hoechst and DRAQ7 and imaged using an automatic high-content confocal microscope. The assay readout included average tumor size, total tumor area, and tumor cell killing and their response curves. We also calculated total tumor area (tumor size × tumor count), which served as a second important endpoint.

### 3.2. Model Development

**Figure 2:**
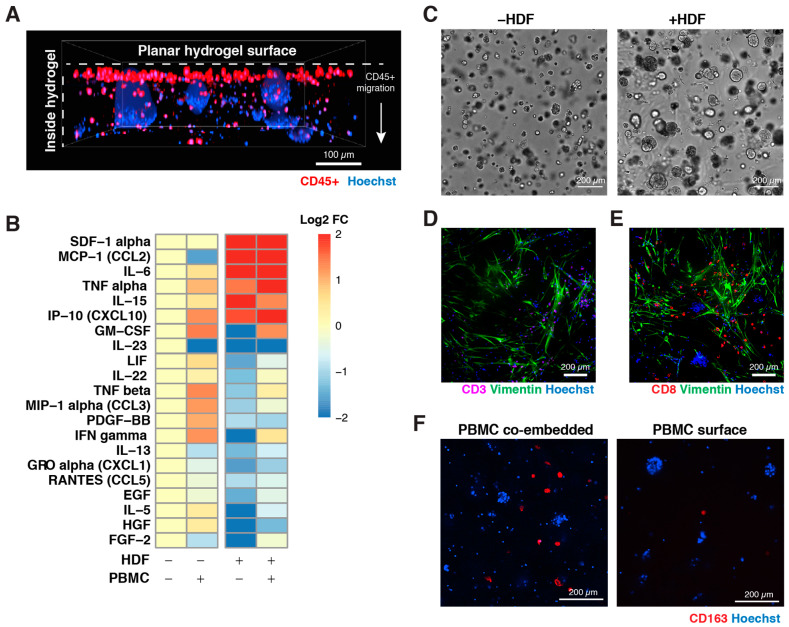
Three-dimensional hydrogel tumor model characterization: (**A**) Immunofluorescence image of a 3D tumor model of CXF1104 with CD45+ cells from PBMCs on the surface of, and infiltrating into, the hydrogel, demonstrating the hydrogel planarity. (**B**) Cytokine analysis comparing key pro-inflammatory markers across the various conditions—with or without HDF, and with or without PBMCs in 3D culture. (**C**) Growth optimization brightfield images of GXA 3067 model without and with HDF in the hydrogel. IF staining for (**D**) CD3 and (**E**) CD8 with vimentin to stain fibroblasts and Hoechst to stain nuclei, and (**F**) CD163 to stain M2 macrophages at endpoint following two 3D model setups—PBMCs co-embedded with tumor and fibroblasts in the hydrogel, and PBMCs seeded on top of the hydrogel surface.

PDX cells and fibroblasts were embedded in flat, thin hydrogels on the bottom of 96-well plates. A proprietary molding technique was utilized during the hydrogel photo-crosslinking step in order to fix the gels in a planar shape, benefiting subsequent PBMC seeding on the surface (Figure 2A) and tumor distribution and growth across the well.

Fetal human dermal fibroblasts (HDF) were co-embedded with PDX cells in the hydrogel to mimic the stromal region of the TME, conferring an immunosuppressive cytokine signature (Figure 2B) with elevated levels of IL-6, MCP-1 (CCL2), CXCL10, SDF-1α and TNF-α compared to the PDX-alone condition, suggesting the role of HDFs in immune modulation.

Growth image analysis of PDX tumorspheres in the hydrogel revealed differences with and without fibroblasts (Figure 2C, Supplementary Figure S1). In some cases, fibroblasts enabled larger tumorspheres to develop (e.g., GXA 3067), while in many of the tumors, the change in tumor size was nominal.

Further characterization of the models via immunofluorescence revealed an abundance of CD3+ (Figure 2D) and CD8+ (Figure 2E) T cells in close proximity to fibroblasts (vimentin stained) and the unlabeled tumor cells (blue aggregates). Later, we stained one PDX model with CD163+ for M2 macrophages (Figure 2F), where PBMCs were either co-embedded with PDX and fibroblasts inside VersaGel or seeded on the hydrogel surface.

### 3.3. Screening Small Molecule Chemotherapeutics on Cypre 30 PDX Panel

**Figure 3:**
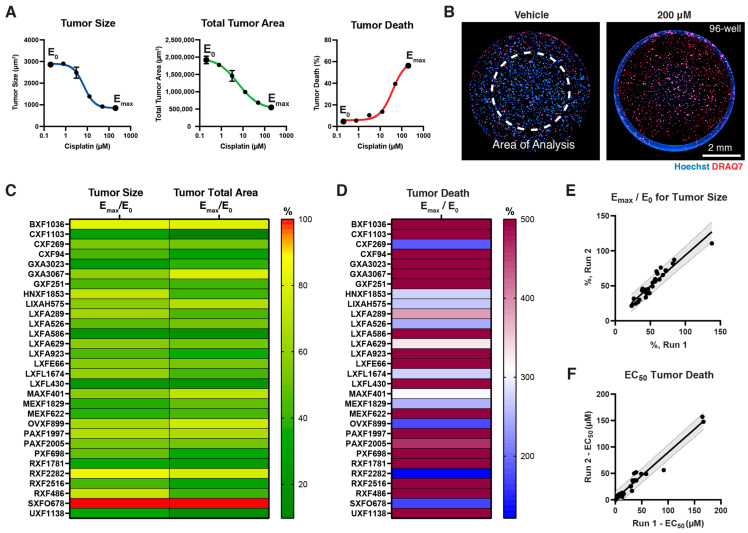
Establishing the 30 Panel analysis: Endpoint analysis of Cisplatin-treated 3D tumor models with PDX and HDF (no PBMCs) in a 6-dose format with the positive (1 µM Staurosporine) and negative controls (vehicle). (**A**) Representative IC50 curves for CXF1103 model for tumor size, total area, and tumor death; (**B**) representative 2× fluorescent images of a well with CXF1103 for vehicle and high-dose 200 µM Cisplatin treatment; (**C**) heat maps for E_max_/E_0_ % for tumor size or total tumor area; (**D**) E_max_/E_0_ % heat map for tumor death; comparison of two screening runs for (**E**) E_max_/E_0_ % of tumor size and (**F**) EC50 of tumor cell death.

Cisplatin was used as the proof-of-concept compound to develop and screen the 30 PDX Panel. Cisplatin is an FDA-approved platinum-based antineoplastic agent that binds to DNA to inhibit cell division. The 30 3D-PDX in vitro models were treated with 6 doses of Cisplatin, with a highest dose of 200 μM and 1:4 serial dilution, for a duration of 4 days. A representative IC50 curve for tumor size, area and death is shown in Figure 3A for the colon line, CXF 1103, along with corresponding high-content images of nuclear (Hoechst) and cell death (DRAQ7) stains (Figure 3B). E_max_/E_0_ values, that is, the maximum response divided by the minimum response on the IC50 curve, were determined for all three values and are shown as a heat map in Figure 3C,D. Supplementary Table S1 details theses values. Notably, many of the compounds responded to Cisplatin treatment by virtue of tumor size/area reduction and cell death increase. To confirm the consistency of the assay, we ran the 30 Panel twice and demonstrated repeatability in both E_max_/E_0_ values (Figure 3E) and EC50 values (Figure 3F). The IC50 range for tumor size reduction, as shown in Supplementary Table S1, was about 0.1 µM to 20 µM across the 30 models; the range for total tumor area was 0.2–40 µM; and the range of EC50 values for cell death was 0.8–167 µM. Notably, the Cmax value for Cisplatin, i.e., the maximum plasma concentration in a patient’s blood, was in the range of 30 µM [19]. Thus, the doses tested were in a clinically relevant window.

### 3.4. Screening Immuno-Oncology Antibodies on Cypre 30 PDX Panel

**Figure 4:**
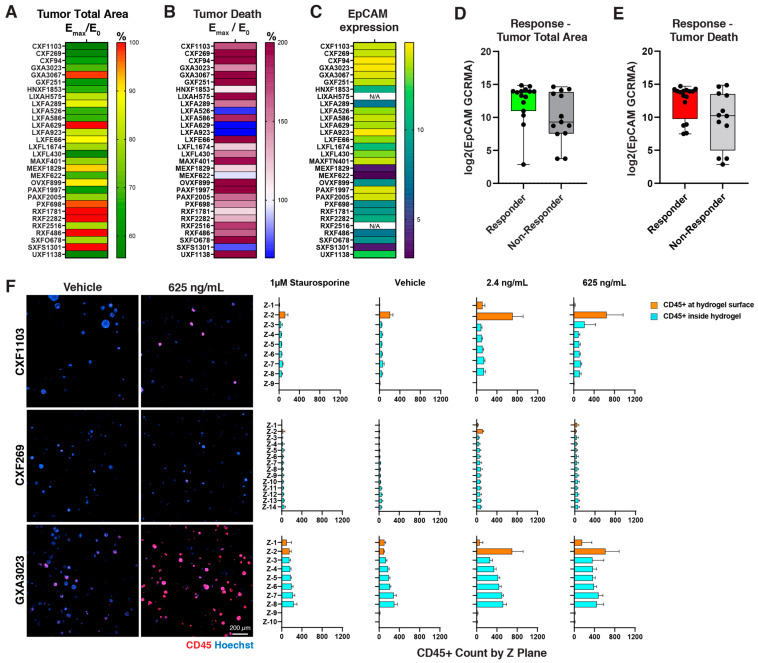
Solitomab immunotherapy screening of Tumor Panel: Endpoint analysis of Solitomab-treated 3D tumor models with PDX, HDF, and PBMCs in a 6-dose format. (**A**) Heat map showing the relative response rates of the 30 in vitro models—calculated values for E_max_/E_0_ for both % tumor area reduction and (**B**) % cell death increase (right). (**C**) Heat map of EpCAM expression (where available) for the 30 Panel; comparison of EpCAM gene expression between the non-responder and responder lines for (**D**) tumor total area and (**E**) tumor death. (**F**) Representative images of CD45 immunofluorescence staining (red) for immune cells in three EpCAM+ high-expression models—CXF1103 (colon), CXF269 (colon) and GXA3067 (gastric), and subsequent Z-plane analysis of CD45+ infiltration into the hydrogel for vehicle, positive control (Staurosporine), and low- (2.4 ng/mL) and high-dose Solitomab (625 ng/mL).

Following its establishment and demonstration of repeatability, we leveraged the 30 in vitro PDX Panel to screen immuno-modulatory antibodies with PBMCs. Three-dimensional tumor models were first grown for the requisite 3–5 days as previously stated, after which compounds and PBMCs were added on the surface of the hydrogel. Due to the gel’s planarity, it allowed for even distribution of the PBMCs and their subsequent immune cell infiltration into the gel to localize around the tumorsphere targets. Solitomab, a bispecific antibody that both activates effector T cells via CD3 and targets tumor surface antigen EpCAM, was screened in a manner similar to that for Cisplatin—6-dose, serially diluted curves with a 4-day treatment duration and high-content imaging endpoints of total tumor area reduction and tumor killing. Results showed that 18 out of 30 PDX models responded to Solitomab, as determined by E_max_/E_0_ tumor total area decreases greater than 20% (Figure 4A, Supplementary Table S2) or tumor cell death increases greater than 50% (Figure 4B, Supplementary Table S2). Where available from the Charles River Cancer Model Database, EpCAM expression was shown in a heat map (Figure 4C). Subsequent plots of responders in terms of tumor area reduction (Figure 4D) and tumor death increase (Figure 4E) revealed a correlation between responder status and EpCAM expression; however, this was not statistically significant. Indeed, some overlap was observed between non-responders and responders for EpCAM expression, potentiating alternative pathways for response or resistance to the CD3/EpCAM bispecific.

We then assessed immune infiltration into the 3D tumor models via immunofluorescence staining in situ. CD45 fluorescent image analysis was performed on three representative tumor models with high EpCAM expression—CXF1103, CXF269 and GXA3067—with Z-plane high-content confocal analysis of infiltrating cells (Figure 4F). Interestingly CXF1103 (microsatellite instability, MSI, high tumor) demonstrated an expansion but sequestration of immune cells toward the surface of the hydrogel; CXF269 (MSI-low) demonstrated low immune infiltration regardless of treatment; and GXA3067 revealed a large expansion and infiltration into the 3D hydrogel model.

**Figure 5:**
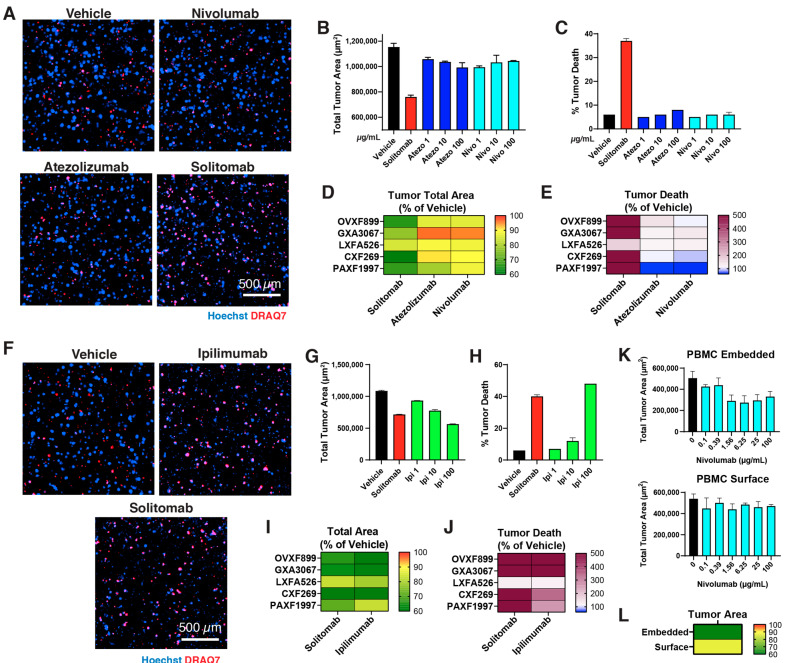
Immune checkpoint inhibitor screening of select models: (**A**) OVXF899 (ovarian) model representative fluorescent images of Hoechst (blue) and DRAQ7 cell death marker (red) taken at endpoint of the four-day assay with PBMCs in response to Nivolumab (anti-PD1) and Atezolizumab (anti-PDL1) compared to vehicle and Solitomab positive control. (**B**) Total tumor area and (**C**) tumor cell death high-content analysis of OVXF899 in 3-dose format for the same. Heat maps of 5 models—OVXF899, GXA3067, LXFA526, CXF269, PAXF1997—for the maximum (**D**) % tumor area reduction and (**E**) % tumor cell death increase compared to the vehicle control. (**F**–**J**) A second screening of ICIs with Ipilimumab (anti-CTLA4), with (**F**) representative images and 3-dose curves for (**G**) tumor area and (**H**) tumor death in OVXF899. Subsequent heat maps for the 5 models with Ipilimumab for maximum (**I**) % tumor area and (**J**) % tumor death compared to the vehicle control. (**K**) Six-dose curves of Nivolumab response in LXFA526 for two PBMC methods (co-embedded vs. surface) and (**L**) the supporting heat map for E_max_/E_0_ % for total tumor area reduction.

In a second round of immunotherapy screening, we assessed anti-tumor responses to immune checkpoint inhibitors (ICIs) in a subset of the models from the 30 Panel (Figure 5). Five models were chosen to analyze tumor area and killing in response to ICIs across a range of indications—OVXF899 (ovarian), GXA3067 (gastric), LXFA526 (NSCLC), CXF269 (colon), and PAXF1997 (pancreatic). OVXF899 was used for representative images (Figure 5A,F) and dose–response analyses against Atezolizumab (anti-PDL1, Atezo), Nivolumab (anti-PD1, Nivo) (Figure 5B–E, Supplementary Table S3), and Ipilimumab (anti-CTLA4, Ipi) (Figure 5G–J, Supplementary Table S4). Solitomab served as the positive control for these experiments, with a different PBMC donor from the previous Solitomab screen. The results showed that Nivolumab and Atezolizumab responded mildly, with tumor area reduction between 0–21% for all models and <150% increase in tumor death compared to the Solitomab positive control, which demonstrated robust tumor area reduction (15–40%) and tumor death increase (174–1318%) in relation to the vehicle (Figure 5D,E). However, OVXF899 did indicate a limited dose-dependent response to Atezolizumab (Figure 5B,C). We subsequently screened Ipilimumab on the same models using the same PBMC donor as that for the first set of ICIs. OVXF899 this time demonstrated a strong dose response to Ipilimumab in both tumor area reduction (Figure 5G) and tumor death increase (Figure 5H). More broadly, Ipilimumab responded in a strong manner similarly to the positive control, Solitomab, across the five models, decreasing tumor area by 17–50% and increasing tumor death by 122–1009% (Figure 5I,J) compared to the vehicle control.

We finally sought to screen one model, LXFA526, in a new 3D assay method by co-embedding the PBMCs with tumor and fibroblasts prior to treatment. We hypothesized this co-encapsulation event may increase the success rate of anti-PD1 and anti-PDL1 due to the immune cells’ increased antigen exposure. Upon screening the two methods with Nivolumab (anti-PD1), we found that co-embedding PBMCs elicited a 39% response in the tumor area reduction compared to 13% reduction for surface-bound PBMCs (Figure 5K–L, Supplementary Table S5).

## 4. Discussion

While prior 3D in vitro models have contributed to the advancement of our study and assays of the TME—such as spheroids revealing multicellular structures that display hypoxia and matrix-based assays such as collagen or basement membrane extract (BME) (static culture or microfluidics based) enabling the evaluation of tumor invasion and gel contraction, these earlier technologies have faced challenges with the incorporation of the stromal and immune compartments of the TME for robust, scalable screening of immunotherapy. In addition, the cell source in a given in vitro assay is paramount, and thus the utility of patient-derived tumor materials in low passage format, as opposed to commercial cell lines, which often derive from a single immortalized cell, is key to recreating the native tumor tissue heterogeneity [20]. The presence of stromal cells, including fibroblasts and immune cells, is a key feature of TME in vivo. It is well-known that cancer-associated fibroblasts (CAFs) can influence cancer initiation and progression through autocrine cytokines/growth factors and modulating extracellular matrix (ECM) [21,22]. Moreover, fibroblasts secrete growth factors and cytokines that can enhance tumor growth [23,24].

Here, we created a novel, hydrogel-based screening panel of solid tumor PDX in vitro models that included the tumor, stromal and immune cells embedded in a patterned, growth-factor-free, planar (flat) ECM hydrogel amenable to small and large molecule diffusion. The VersaGel hydrogel patterning technology was successfully employed for culturing patient-derived tumors and PDX cells previously [17]. The tumor panel comprised 30 PDX models of various tumor indications (NSCLC, colon, renal, melanoma, breast, pancreatic, gastric, etc.) to screen oncology and immuno-oncology therapies in a dose-dependent manner.

PDX-derived cell lines, as opposed to PDX ex vivo tissue fragments, were ultimately employed in these tumor panels for their ability to expand in vitro up to 10 passages, which opened the door for multiple screening runs with reproducible data, whereas ex vivo tissue had limited expansion potential for scaled-up, let alone repeated, screening. In other studies, groups have successfully used PDX and PDX organoids in vitro for screening of targeted therapies and immunotherapy [25]. It is in the authors’ opinion that extensive expansion of these organoids is first required prior to screening, and moreover, their co-culture with fibroblasts and analysis of immune cell infiltration through the ECM and fibroblast-enriched stroma can be challenging, and the platform described here solves for both.

From afar, the use of HDF may limit the ability to recreate the cancer-associated fibroblast phenotype of the TME. In our analysis of cytokines from the 3D model supernatants, however, several critical markers of an immunosuppressive tumor microenvironment were demonstrated when culturing HDF with tumor cells (versus tumor alone), as evidenced by the upregulation of IL6, MCP-1, and SDF-1a. Moreover, neonatal HDFs were previously determined to contribute to the crosstalk with lung cancer cells in a capacity similar to that of CAF cells, and specifically, in their induction of NFκB signaling [26]. Further analysis may be warranted, but these data suggest a promising comparison between HDF and CAF when co-cultured with tumor cells. Indeed, it would be interesting to provide a comparison of HDF and CAF in the context of the current assay endpoints, such as immune migration and T cell-mediated killing in response to checkpoint inhibitors.

It should also be noted that the use of non-HLA or donor-matched PBMCs and fibroblasts may present a potential concern with alloreactivity. Previous research suggested that humanized mice require a therapeutic window within ~4 weeks of PBMC implantation before the onset of graft versus host disease (GVHD) [27,28]. Thus, we concluded that a 4–7 day window for our in vitro experiments would show negligible signs of GVHD. Moreover, the proper controls of testing a vehicle control in every experiment were utilized to inform the baseline tumor size, area, and killing percentages as compared to the dose–response curves for Solitomab, Nivolumab, Atezolizumab, and Ipilimumab. Future experiments may reveal segmentation of alloreactivity based on the immune cell phenotype (e.g., CD3, CD8).

The robust response to Ipilimumab (anti-CTLA4) and mild responses to Nivolumab (anti-PD1) and Atezolizumab (anti-PDL1) in the 3D assays were investigated in the context of the broader literature. First, CTLA4 regulates T-cell activation at the earliest stage in the lymph node, whereas PD1 limits T cells later in the tumor tissue [29]. This may help to explain why Ipilimumab demonstrated significant anti-tumor responses in the 3D assays when PBMCs were seeded on the hydrogel surface and cultured for 4 days, as the PBMCs were transitioning from naïve to early activation. PD1 expression, on the other hand, increases during chronic antigen exposure in the TME [30], and thus, 4 days of culture duration coupled with a physical separation of immune and tumor cells in the seeding approach may be rate-limiting factors of the surface seeding approach.

In one study, tumors were stimulated with IFNg prior to culture with PBMCs and anti-PD1, and this process was repeated for several weeks to generate tumor-reactive T cells with a demonstrated killing capacity [31]. Other studies showed robust responses to anti-PD1 by utilizing dissociated tumor fragments ex vivo from syngeneic mice or patient-derived samples, with the implication that tumor-infiltrating lymphocytes (TILs) within these tissues responded to the treatment due to their PD1+ expression and tumor reactivity compared to naïve PBMCs [32,33]. These studies suggest that both timing and exposure to PBMC stimulation with antigens and IFNγ may be a nuanced balance in order to demonstrate responses to PD1/PDL1 inhibitors. Our final experiment of co-embedding PBMCs with tumor cells prior to anti-PD1 treatment promoted this concept that antigen exposure and spatial proximity to tumor cells may indeed be critical to “training” the tumor-reactive immune cell populations; however, further investigations are needed to support this hypothesis [30].

In conclusion, the present study demonstrates a novel way to recreate the tumor microenvironment by co-culturing PDX tumor cells, stromal fibroblasts, and immune cells (PBMCs) in a planar 3D hydrogel for robust and repeatable screening via high-content image analysis. The platform enables both oncology and immuno-oncology therapy screening in a dose-dependent manner and showcases the ability to retain key immune phenotypes such as T cells (CD4, CD8) and myeloid cells (e.g., M2 macrophages). Moreover, the platform enables Z-stack analysis of T-cell infiltration into the hydrogel when PBMCs are seeded on the gel surface and allows the assessment of immuno-modulatory compounds such as anti-CD3/EpCAM and immune checkpoint inhibitors such as anti-PD1/PDL1 and anti-CTLA4. In the future, it will be important to assess the key differences of non-matched, HLA-matched, and patient-matched immune cell populations in the 3D system, as well as the TILs versus surface-bound PBMCs for assaying anti-PD1 response. In addition, ideally, the direct comparison of patient response data with the 3D ex vivo outcomes in the platform would provide the most substantial route to translational relevance. Finally, the prospect of generating tumor-reactive immune populations ex vivo may open the door for novel methods in adoptive cell transfer. The drug discovery industry is entering a new age where in vitro and ex vivo platforms are becoming increasingly utilized to reduce and replace animal studies and spur the innovation of novel therapeutics targeting the TME.

## Figures and Tables

**Table 1:** 3D in vitro PDX Models used in the Tumor Panel.

Cancer Type	Tumor Designation	Model #	Histology	Primary/Metastasis/Recurrent
Bladder *	BXF	1036	urothelial carcinoma	Metastasis
Colon	CXF	94	adenocarcinoma	Primary
Colon	CXF	269	adenocarcinoma	Primary
Colon	CXF	1103	adenocarcinoma	Metastasis
Gastric	GXA	3023	adenocarcinoma Lauren intestinal type	Primary
Gastric	GXA	3067	adenocarcinoma	Primary
Gastric *	GXF	251	adenocarcinoma	Primary
Head neck	HNXF	1853	squamous cell carcinoma	Recurrent
Liver *	LIXFC	2050	cholangiocarcinoma	Primary
Liver	LIXFC	575	cholangiocarcinoma	Primary
NSCLC (adeno)	LXFA	289	adenocarcinoma	Metastasis
NSCLC (adeno)	LXFA	526	adenocarcinoma	Metastasis
NSCLC (adeno)	LXFA	586	adenocarcinoma	Primary
NSCLC (adeno)	LXFA	629	adenocarcinoma	Primary
NSCLC (adeno)	LXFA	923	adenocarcinoma	Metastasis
NSCLC (epidermoid)	LXFE	66	squamous cell carcinoma	Primary
NSCLC (large cell)	LXFL	1674	large cell carcinoma	Primary
NSCLC (large cell)	LXFL	430	large cell carcinoma	Primary
Breast (Triple Negative)	MAXFTN	401	adenocarcinoma	Metastasis
Melanoma	MEXF	1829	melanoma	Metastasis
Melanoma	MEXF	622	melanoma	Metastasis
Ovarian	OVXF	899	serous adenocarcinoma	Primary
Pancreatic	PAXF	1997	adenocarcinoma	Primary
Pancreatic	PAXF	2005	adenocarcinoma	Primary
Mesothelioma	PXF	698	pleural mesothelioma	Primary
Renal	RXF	1781	clear cell carcinoma	Recurrent
Renal	RXF	2282	clear cell carcinoma	not known
Renal	RXF	2516	clear cell carcinoma	not known
Renal	RXF	486	hypernephroma	Primary
Osteosarcoma	SXFO	678	osteosarcoma	Primary
Soft tissue sarcoma	SXFS	1301	rhabdomyosarcoma	Metastasis
Uterus	UXF	1138	sarcoma	Metastasis

* Note: LIXFC2050 was used in the 30 Panel for Cisplatin and was replaced with GXF251 for Solitomab screening. BXF 1036 was added to the Panel for Cisplatin screening to replace SXFS 1301. Model # corresponds to PDX annotation provided on Charles River’s Cancer Model Database.

## Data Availability

The data presented in this study are available in the supplementary material.

## Supplementary Materials

The following supporting information can be downloaded at: https://www.mdpi.com/article/10.3390/cells12081145/s1, Figure S1: Tumor Sizes in Hydrogel; Table S1: Cisplatin data on 30 Panel; Table S2: Solitomab data on 30 Panel; Table S3: Atezolizumab, Nivolumab Data; Table S4: Ipilimumab Data; Table S5: Nivolumab data using two PBMC seeding methods.

## References

[1] Turley S.J., Cremasco V., Astarita J.L. (2015). Immunological Hallmarks of Stromal Cells in the Tumour Microenvironment. Nat. Rev. Immunol..

[2] Balkwill F.R., Capasso M., Hagemann T. (2012). The Tumor Microenvironment at a Glance. J. Cell Sci..

[3] Anderson N.M., Simon M.C. (2020). The Tumor Microenvironment. Curr. Biol..

[4] Kather J.N., Suarez-Carmona M., Charoentong P., Weis C.-A., Hirsch D., Bankhead P., Horning M., Ferber D., Kel I., Herpel E. (2018). Topography of Cancer-Associated Immune Cells in Human Solid Tumors. eLife.

[5] Whiteside T.L. (2008). The Tumor Microenvironment and Its Role in Promoting Tumor Growth. Oncogene.

[6] Mariathasan S., Turley S.J., Nickles D., Castiglioni A., Yuen K., Wang Y., Kadel E.E., Koeppen H., Astarita J.L., Cubas R. (2018). TGFβ Attenuates Tumour Response to PD-L1 Blockade by Contributing to Exclusion of T Cells. Nature.

[7] Nicolas-Boluda A., Vaquero J., Vimeux L., Guilbert T., Barrin S., Kantari-Mimoun C., Ponzo M., Renault G., Deptula P., Pogoda K. (2021). Tumor Stiffening Reversion through Collagen Crosslinking Inhibition Improves T Cell Migration and Anti-PD-1 Treatment. eLife.

[8] Thurber G.M., Schmidt M.M., Wittrup K.D. (2008). Antibody Tumor Penetration: Transport Opposed by Systemic and Antigen-Mediated Clearance. Adv. Drug Deliv. Rev..

[9] Cancer Cell Line Encyclopedia Consortium, Genomics of Drug Sensitivity in Cancer Consortium (2015). Pharmacogenomic Agreement between Two Cancer Cell Line Data Sets. Nature.

[10] Danku A.E., Dulf E.-H., Braicu C., Jurj A., Berindan-Neagoe I. (2022). Organ-on-a-Chip: A Survey of Technical Results and Problems. Front. Bioeng. Biotechnol..

[11] Ingber D.E. (2022). Human Organs-on-Chips for Disease Modelling, Drug Development and Personalized Medicine. Nat. Rev. Genet..

[12] Yakavets I., Francois A., Benoit A., Merlin J.-L., Bezdetnaya L., Vogin G. (2020). Advanced Co-Culture 3D Breast Cancer Model for Investigation of Fibrosis Induced by External Stimuli: Optimization Study. Sci. Rep..

[13] Edwards S.J., Carannante V., Kuhnigk K., Ring H., Tararuk T., Hallböök F., Blom H., Önfelt B., Brismar H. (2020). High-Resolution Imaging of Tumor Spheroids and Organoids Enabled by Expansion Microscopy. Front. Mol. Biosci..

[14] Vukicevic S., Kleinman H.K., Luyten F.P., Roberts A.B., Roche N.S., Reddi A.H. (1992). Identification of Multiple Active Growth Factors in Basement Membrane Matrigel Suggests Caution in Interpretation of Cellular Activity Related to Extracellular Matrix Components. Exp. Cell Res..

[15] Zhu Y.K., Umino T., Liu X.D., Wang H.J., Romberger D.J., Spurzem J.R., Rennard S.I. (2001). Contraction of Fibroblast-Containing Collagen Gels: Initial Collagen Concentration Regulates the Degree of Contraction and Cell Survival. Vitr. Cell. Dev. Biol. Anim..

[16] Roth T., Burger A.M., Dengler W., Willmann H., Fiebig H.H. (1999). Human Tumor Cell Lines Demonstrating the Characteristics of Patient Tumors as Useful Models for Anticancer Drug Screening. Contrib. Oncol..

[17] Hribar K.C., Wheeler C.J., Bazarov A., Varshneya K., Yamada R., Buckley P., Patil C.G. (2019). A Simple Three-Dimensional Hydrogel Platform Enables Ex Vivo Cell Culture of Patient and PDX Tumors for Assaying Their Response to Clinically Relevant Therapies. Mol. Cancer Ther..

[18] (2016). N-Parameter Logistic Regression.

[19] Rajkumar P., Mathew B.S., Das S., Isaiah R., John S., Prabha R., Fleming D.H. (2016). Cisplatin Concentrations in Long and Short Duration Infusion: Implications for the Optimal Time of Radiation Delivery. J. Clin. Diagn. Res..

[20] Richter M., Piwocka O., Musielak M., Piotrowski I., Suchorska W.M., Trzeciak T. (2021). From Donor to the Lab: A Fascinating Journey of Primary Cell Lines. Front. Cell Dev. Biol..

[21] Bhowmick N.A., Neilson E.G., Moses H.L. (2004). Stromal Fibroblasts in Cancer Initiation and Progression. Nature.

[22] Sahai E., Astsaturov I., Cukierman E., DeNardo D.G., Egeblad M., Evans R.M., Fearon D., Greten F.R., Hingorani S.R., Hunter T. (2020). A Framework for Advancing Our Understanding of Cancer-Associated Fibroblasts. Nat. Rev. Cancer.

[23] Olumi A., Grossfeld G., Hayward S., Carroll P., Cunha G., Hein P., Tlsty T. (2000). Carcinoma-Associated Fibroblasts Stimulate Tumor Progression of Initiated Human Epithelium. Breast Cancer Res..

[24] Orimo A., Gupta P.B., Sgroi D.C., Arenzana-Seisdedos F., Delaunay T., Naeem R., Carey V.J., Richardson A.L., Weinberg R.A. (2005). Stromal Fibroblasts Present in Invasive Human Breast Carcinomas Promote Tumor Growth and Angiogenesis through Elevated SDF-1/CXCL12 Secretion. Cell.

[25] Takahashi N., Higa A., Hiyama G., Tamura H., Hoshi H., Dobashi Y., Katahira K., Ishihara H., Takagi K., Goda K. (2021). Construction of in Vitro Patient-Derived Tumor Models to Evaluate Anticancer Agents and Cancer Immunotherapy. Oncol. Lett..

[26] Rudisch A., Dewhurst M.R., Horga L.G., Kramer N., Harrer N., Dong M., Van Der Kuip H., Wernitznig A., Bernthaler A., Dolznig H. (2015). High EMT Signature Score of Invasive Non-Small Cell Lung Cancer (NSCLC) Cells Correlates with NFκB Driven Colony-Stimulating Factor 2 (CSF2/GM-CSF) Secretion by Neighboring Stromal Fibroblasts. PLoS ONE.

[27] Morillon Y.M., Sabzevari A., Schlom J., Greiner J.W. (2020). The Development of Next-Generation PBMC Humanized Mice for Preclinical Investigation of Cancer Immunotherapeutic Agents. Anticancer Res..

[28] Pearson T., Greiner D.L., Shultz L.D., Coligan J.E. (2008). Creation of “Humanized” Mice to Study Human Immunity. Current Protocols in Immunology.

[29] Buchbinder E.I., Desai A. (2016). CTLA-4 and PD-1 Pathways: Similarities, Differences, and Implications of Their Inhibition. Am. J. Clin. Oncol..

[30] Simon S., Labarriere N. (2018). PD-1 Expression on Tumor-Specific T Cells: Friend or Foe for Immunotherapy?. Oncoimmunology.

[31] Dijkstra K.K., Cattaneo C.M., Weeber F., Chalabi M., van de Haar J., Fanchi L.F., Slagter M., van der Velden D.L., Kaing S., Kelderman S. (2018). Generation of Tumor-Reactive T Cells by Co-Culture of Peripheral Blood Lymphocytes and Tumor Organoids. Cell.

[32] Jenkins R.W., Aref A.R., Lizotte P.H., Ivanova E., Stinson S., Zhou C.W., Bowden M., Deng J., Liu H., Miao D. (2018). Ex Vivo Profiling of PD-1 Blockade Using Organotypic Tumor Spheroids. Cancer Discov..

[33] Aref A.R., Campisi M., Ivanova E., Portell A., Larios D., Piel B.P., Mathur N., Zhou C., Coakley R.V., Bartels A. (2018). 3D Microfluidic Ex Vivo Culture of Organotypic Tumor Spheroids to Model Immune Checkpoint Blockade. Lab Chip.

