# Supplementary material for: A Novel Hydrogel-Based 3D In Vitro Tumor Panel of 30 PDX Models Incorporates Tumor, Stromal and Immune Cell Compartments of the TME for the Screening of Oncology and Immuno-Therapies
Source: Cells. 2023 Apr 13;12(8):1145. doi: 10.3390/cells12081145 (PMC10137152; doi:10.3390/cells12081145)
Supplement: Supplementary file 1 [file cells-12-01145-s001.zip › cells-2245126-supplementary.pdf]

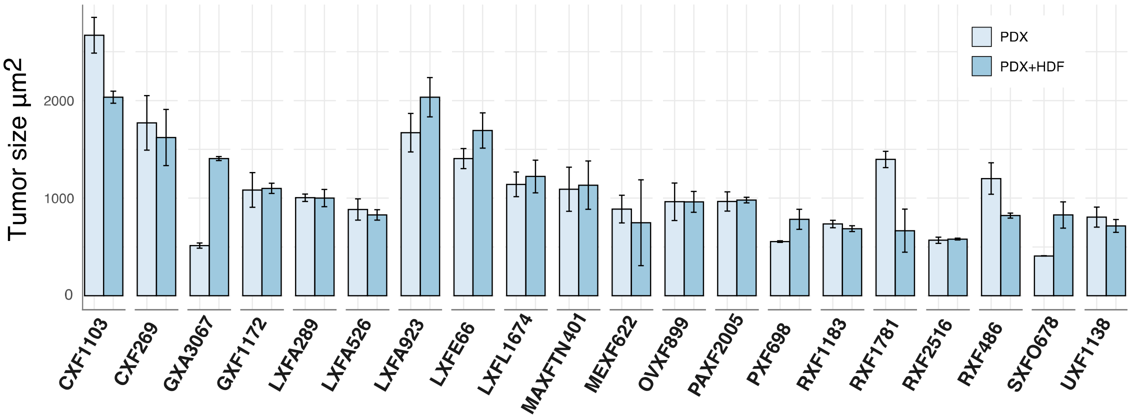

Supplementary Figure S1: Size differences of three-dimensional PDX tumorspheres in the hydrogel with or without fibroblasts (HDF).

Supplementary Table S1: Cisplatin response in the 30 PDX Panel with respect to tumor size, total tumor area, and tumor cell death.

Tumor Size:

PDX	IC50	E0 µm2	Emax µm2	Emax/E0 %	Model
BXF1036	0.410	1,197.304	984.391	82.217	Non-responder
CXF1103	6.702	2,893.946	847.768	29.295	Size decrease
CXF269	3.272	1,932.417	1,033.153	53.464	Size decrease
CXF94	18.871	2,614.970	1,137.931	43.516	Size decrease
GXA3023	1.539	3,489.259	924.289	26.490	Size decrease
GXA3067	19.726	1,632.501	959.381	58.768	Size decrease
GXF251	0.155	1,991.304	874.050	43.893	Size decrease
HNXF1853	12.549	548.885	395.661	72.099	Size decrease
LIXAH575	3.346	1,031.103	585.118	56.747	Size decrease
LXFA289	9.718	1,339.160	845.057	63.104	Size decrease
LXFA526	1.807	1,640.877	768.664	46.845	Size decrease
LXFA586	4.068	1,879.458	458.624	24.402	Size decrease
LXFA629	1.930	2804.442	1670.827	59.578	Size decrease
LXFA923	5.392	1,800.854	807.267	44.827	Size decrease
LXFE66	1.112	1,640.863	934.159	56.931	Size decrease
LXFL1674	0.542	1,463.166	880.382	60.170	Size decrease
LXFL430	1.135	2020.332	431.899	21.378	Size decrease
MAXF401	3.839	1,653.390	952.089	57.584	Size decrease
MEXF1829	0.628	1,514.724	577.630	38.134	Size decrease
MEXF622	0.131	1,642.478	640.231	38.980	Size decrease
OVXF899	2.071	1,089.333	708.005	64.994	Size decrease
PAXF1997	12.830	1,057.049	718.323	67.956	Size decrease
PAXF2005	0.745	1,172.634	623.795	53.196	Size decrease
PXF698	6.530	1,110.793	545.025	49.066	Size decrease
RXF1781	12.657	1,493.652	517.138	34.622	Size decrease
RXF2282	11.290	901.520	759.762	84.276	Non-responder
RXF2516	1.975	1,510.002	653.449	43.275	Size decrease
RXF486	14.036	1348.326	966.731	71.699	Non-responder
SXFO678	12.930	799.479	1,102.661	137.922	Size increase
UXF1138	4.298	2,470.256	802.779	32.498	Size decrease

Cut-off value

Size decrease: E_{max}/E_0 % <= 80%

Non-responder: E_{max}/E_0 % > 80%

Total Tumor Area:

PDX	IC50	E0 µm2	Emax µm2	Emax/E0 %	Model
BXF1036	3.147	1,389,173.684	1,135,661.811	81.751	Non-responder
CXF1103	5.137	2,023,381.740	551,077.390	27.235	Total area decrease
CXF269	2.055	1,151,246.537	455,655.324	39.579	Total area decrease
CXF94	19.989	1,531,522.931	713,386.558	46.580	Total area decrease
GXA3023	1.077	1,424,361.712	357,845.792	25.123	Total area decrease
GXA3067	18.249	1,901,372.440	1,295,057.146	68.112	Total area decrease
GXF251	0.280	1,299,385.648	543,685.155	41.842	Total area decrease
HNXF1853	3.808	719,113.042	304,108.029	42.289	Total area decrease
LIXAH575	2.310	1,217,517.701	773,179.791	63.505	Total area decrease
LXFA289	40.067	1,117,475.276	606,288.142	54.255	Total area decrease
LXFA526	2.233	1,085,465.113	476,134.625	43.865	Total area decrease
LXFA586	4.852	1,639,872.286	254,047.076	15.492	Total area decrease
LXFA629	3.034	1,042,007.864	508,655.491	48.815	Total area decrease
LXFA923	14.922	1,293,031.686	513,748.508	39.732	Total area decrease
LXFE66	1.774	1,422,400.821	823,195.947	57.874	Total area decrease
LXFL1674	2.177	1,431,322.643	861,936.070	60.220	Total area decrease
LXFL430	0.965	1,128,632.246	260,115.587	23.047	Total area decrease
MAXF401	5.265	1,822,982.132	1,072,091.503	58.810	Total area decrease
MEXF1829	0.741	1,278,186.541	453,558.531	35.485	Total area decrease
MEXF622	0.188	1,208,668.882	460,941.281	38.136	Total area decrease
OVXF899	1.954	1,357,645.255	909,396.612	66.983	Total area decrease
PAXF1997	4.025	1,179,388.292	678,701.389	57.547	Total area decrease
PAXF2005	0.463	686,066.241	270,225.903	39.388	Total area decrease
PXF698	11.591	687,747.327	404,327.741	58.790	Total area decrease
RXF1781	15.222	1,240,237.511	436,083.384	35.161	Total area decrease
RXF2282	25.552	1,178,304.752	1,007,499.161	85.504	Non-responder
RXF2516	7.278	1,110,121.734	453,792.124	40.878	Total area decrease
RXF486	4.206	623,394.394	276,094.022	44.289	Total area decrease
SXFO678	0.831	904,502.296	1,350,703.010	149.331	Total area increase
UXF1138	0.832	1,301,241.870	436,428.211	33.539	Total area decrease

Cut-off value

Total area decrease: E_{max}/E_0 % <= 80%

Non-responder: E_{max}/E_0 % > 80%

Tumor Death:

PDX	EC50	E0 %	Emax %	Emax/E0 %	Model
BXF1036	4.606	8.596	37.198	432.760	Death increase
CXF1103	35.510	7.215	56.282	780.051	Death increase
CXF269	N/A	1.367	2.832	207.097	N/A
CXF94	165.120	7.448	28.454	382.034	Death increase
GXA3023	31.550	10.969	43.842	399.684	Death increase
GXA3067	36.776	2.973	31.683	1,065.787	Death increase
GXF251	6.588	4.415	22.342	506.007	Death increase
HNXF1853	13.308	4.710	12.967	275.310	Death increase
LIXAH575	4.565	12.653	32.118	253.841	Death increase
LXFA289	167.137	12.166	34.487	283.471	Death increase
LXFA526	3.726	10.970	18.171	165.642	Death increase
LXFA586	3.982	9.837	54.845	557.551	Death increase
LXFA629	49.859	12.896	39.271	304.513	Death increase
LXFA923	91.761	0.884	16.232	1,836.260	Death increase
LXFE66	9.015	5.801	47.390	816.966	Death increase
LXFL1674	12.258	13.007	25.241	194.055	Death increase
LXFL430	7.890	6.466	59.187	915.398	Death increase
MAXF401	39.812	11.830	46.283	391.230	Death increase
MEXF1829	16.027	14.435	49.451	342.574	Death increase
MEXF622	3.056	5.198	56.110	1,079.498	Death increase
OVXF899	3.194	13.133	29.501	224.630	Death increase
PAXF1997	58.423	1.227	7.386	602.204	Death increase
PAXF2005	7.982	6.104	41.309	676.784	Death increase
PXF698	32.655	3.123	21.643	692.936	Death increase
RXF1781	28.964	6.543	52.770	806.552	Death increase
RXF2282	0.819	28.293	24.909	88.038	Death decrease
RXF2516	39.426	3.046	38.636	1,268.221	Death increase
RXF486	33.690	9.260	65.183	703.888	Death increase
SXFO678	13.038	23.720	35.002	147.567	Death increase
UXF1138	29.566	3.430	42.691	1,244.711	Death increase

Cut-off value

Death decrease: $E_{max}>E_0$

Death increase: $E_{max}<E_0$

Supplementary Table S2: Solitomab E_{max}/E_0 % in the 30 PDX Panel for total tumor area and death

PDX	Tumor Area	Tumor Death
CXF1103	55.47	167.577
CXF269	57.536	211.625
CXF94	64.349	258.689
GXA3023	70.387	146.802
GXA3067	97.648	526.834
GXF251	62.026	282.019
HNXF1853	68.037	108.797
LIXAH575	81.715	197.973
LXFA289	86.913	155.412
LXFA526	68.601	70.791
LXFA586	68.575	184.121
LXFA629	107.446	64.752
LXFA923	83.035	63.279
LXFE66	89.734	704.046
LXFL1674	78.708	120.973
LXFL430	57.981	152.877
MAXF401	77.553	193.14
MEXF1829	91.838	117.033
MEXF622	69.16	95.368
OVXF899	90.761	203.143
PAXF1997	63.019	268.438
PAXF2005	76.796	158.448
PXF698	96.807	146.05
RXF1781	101.658	116.331
RXF2282	99.742	131.239
RXF2516	79.691	176.251
RXF486	110.947	162.793
SXFO678	78.699	48210.8
SXFS1301	117.859	75.287
UXF1138	57.175	1173.58

Supplementary Table S3: ICI Atezolizumab and Nivolumab heat maps of total tumor area and tumor cell death compared to the positive control, Solitomab, in a subset up PDX models.

E_{max}/E_0 % for Total Tumor Area

PDX	Solitomab	Atezolizumab	Nivolumab
OVXF 899	66	86	86
GXA 3067	77	96	95
LXFA 526	85	89	88
CXF 269	60	91	90
PAXF 1997	67	79	88

Solitomab served as the positive control.

E_{max}/E_0 % for Tumor Death

PDX	Solitomab	Atezolizumab	Nivolumab
OVXF 899	587	147	98
GXA 3067	1262	118	136
LXFA 526	174	115	121
CXF 269	1318	98	90
PAXF 1997	617	67	65

Solitomab served as the positive control.

Supplementary Table S4: ICI Ipilimumab heat maps of total tumor area and tumor cell death compared to the positive control, Solitomab, in a subset up PDX models.

E_{max}/E_0 % for Total Tumor Area

PDX	Solitomab	Ipilimumab
OVXF 899	66	52
GXA 3067	64	61
LXFA 526	83	79
CXF 269	52	50
PAXF 1997	71	83

Solitomab served as the positive control.

E_{max}/E_0 % for Tumor Death

PDX	Solitomab	Ipilimumab
OVXF 899	669	794
GXA 3067	1195	1009
LXFA 526	126	122
CXF 269	1796	341
PAXF 1997	1053	265

Solitomab served as the positive control.

Supplementary Table S5: PBMCs embedded vs surface E_{max}/E_0 % for Total Tumor Area for one PDX model, LXFA 526, with ICI Nivolumab treatment

PDX	Total Tumor Area
Embedded	61
Surface	87

The same PBMC was used for both the embedding and surface test.
